# Practical counting of substitutive paths on a planar infrastructure network

**DOI:** 10.1038/s41598-022-18927-w

**Published:** 2022-08-29

**Authors:** Yukio Hayashi, Atsushi Tanaka

**Affiliations:** 1 Japan Advanced Institute of Science and Technology, Graduate School of Advanced Institute of Science and Technology, Nomi, 923-1292 Japan; 2 Yamagata University, Graduate School of Science and Engineering, Yonezawa, 992-8510 Japan

**Keywords:** Complex networks, Applied mathematics, Computer science, Engineering

## Abstract

When there are many non-intersecting paths between two vertices on a network, the connectivity is fault-tolerant. Because of no common vertices on these paths, they can be emergently used in avoiding destroyed parts on the usual paths by any disasters or attacks. It gives a tolerance index whether the combination of non-intersecting paths is many or few. However, to enumerate such paths is an intractable combinatorial problem, no practical algorithm has been known. On the other hand, many socio-technological infrastructure networks are embedded on the surface of Earth. Thus, as an approximate solution, we extendedly apply the counting method based on a path matrix with our proposed mapping to directed acyclic graphs from a planar network according to each pair of source and terminal vertices. The tendency of many or few combinations of the paths is clearly investigated through computer simulations for realistic networks. This approach will be useful for evaluating the existence of substitutive paths to improve the tolerance in risk management.

## Introduction

The resilience has attracted much attention pointed out as follows^1^. The state-of-the-art in the study of the resilience of complex systems is to see resilience as the ability to withstand and recover from some specific shock^1–3^. The ability to withstand shocks is often referred to as robustness^4,5^ and is often seen as a structural component of the resilience of a system. In this case, it could correspond to the connectivity of the network through non-intersecting paths. The ability to recover basic function of networking from shocks is instead a dynamical property^6,7^. It requires time to be accounted for and is not part of the study in this article. For example, in this case, it could refer to the time it takes the network to switch to an alternative available path or create a new one to re-establish connectivity. In other words, if substitutive paths between two vertices exist, they can be emergently used just after unspecified disasters or attacks.

We discuss the robustness of path between two end vertices, whose combination number of substitutive paths is changeable according to the selection of end vertices. Our problem setting for the changeable number differs to discuss the percolation or robustness of whole connectivity against random failures in bicomponent^8^ or component of *k*-connectivity^9^ as a set of vertices such that every pair of vertices has at least 2 or (a fixed) *k* node-disjoint paths. In the previous studies, the importance of edge for disconnecting a network is evaluated by using the ratio cut partitioning^10,11^. Since the minimum ratio cut problem is NP-hard^12^ with the constraint of integer values as the selection number of vertices for partitioning, it is relaxed with real values to analyze the Laplacian of graph. In the approximation, a given graph is divided into two parts by cutting (removing) the most vulnerable minimum edges whose end-vertices have positive and negative signs of the eigenvector’s elements for the second smallest eigenvalue of the Laplacian^13^. However, in this method, we can not discriminate whether substitutive paths between two vertices exist or not. Thus, we aim to investigate the number of non-intersecting paths which give the possibility of passing through undamaged vertices and edges as a measure of bypass effect, when a usually worked path is disconnected by partial removals after any disasters or attacks. Here, non-intersecting paths means that there are no common vertices and edges on each other’s path.

On the other hand, to determine the existence of non-intersecting paths is also called disjoint paths problem in computer science for a given vertex sets of source $\{ s_{1}, s_{2}, \ldots , s_{k} \}$ and terminal $\{ t_{1}, t_{2}, \ldots , t_{k} \}$ in a graph. This problem is known as NP-complete^14^ for a fixed integer variable $k \ge 2$ even in a planar undirected graph. Although only the existence of polynomial-time algorithm has been shown, it is unfamiliar and incomprehensible except for graph theory researchers because of its great length proof (with more than hundred pages in series papers) based on the difficult theorems in graph minors^15^. Moreover, no practical algorithm has been known even for $k = 3$^16^. In a naive method, all combinations of the vertices are checked on *k* candidates of paths to be non-intersecting, this is obviously inefficient and practically intractable with huge computation. Thus, as another approach, we focus on the counting method applied to Young tableaux or plain partitions in mathematical physics related to representation theory or combinatorics. Because we can use a theorem that the combination number of non-intersecting paths is given by the determinant of a path matrix with total positivity^17^. However, since the targets of this counting based on a path matrix are only directed acyclic graphs, we must develop a mapping in order to apply it for counting the number of non-intersecting paths on a given network as undirected planar graph. Our key idea is that the direction of edge is not fixed but temporally assigned for each pair of source and terminal vertices. In application point of view, a reason of considering planar graphs is that many infrastructure networks, such as power-grid, gas-pipeline, water-supply, transportation, and communication systems, are constructed on the surface of Earth, therefore considered as planar graphs conceptually.

## Results

At first, we summarize symbols used in this paper as shown in Table 1.

**Table 1:** Symbol table.

Notation	Description
*N*	Number of vertices
*M*	Number of edges
*k*	Fixed number of non-intersecting paths
*s*	Source vertex for a path
*t*	Terminal vertex for a path
$\partial s$	Set of the neighbors of vertex *s*
$| \partial s |$	Size of $\partial s$
$s_{1}, s_{2}, \ldots , s_{k}$	Some vertices chosen from the nearest or next-nearest neighbors of *s*
$t_{1}, t_{2}, \ldots , t_{k}$	Some vertices chosen from the nearest or next-nearest neighbors of *t*
SI-IT-FR	Path through vertices of countries or other indices SI, IT, and FR in this order
SI $\sim$ FR	Paths between SI and FR
*P*, *Q*	Path between vertices
${{{\mathcal {P}}}}(s_{i}, t_{j})$	Set of paths from $s_{i}$ to $t_{j}$
$w_{ij}$	*i*-*j* element of path matrix *W*
$\det W$	Determinant of matrix *W*
$\Pi _{i} L_{ii}$	Product of the diagonal elements $L_{ii}$ of matrix *L*
$u \rightarrow v$	Vector from *u* to *v*

**Figure 1:**
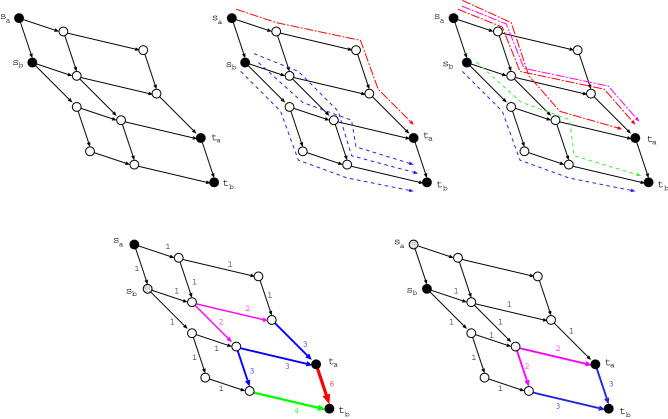
Example of six combinations of two non-intersecting paths from $s_{a}$ to $t_{a}$ and from $s_{b}$ to $t_{b}$. (Top-Left) A planar directed graph with pairs of $k = 2$ boundary vertices. (Top-Middle) One $s_{a}$-$t_{a}$ path colored by red and its corresponding three $s_{b}$-$t_{b}$ paths colored by blue. (Top-Right) Two $s_{b}$-$t_{b}$ paths colored by blue or green and their corresponding $s_{a}$-$t_{a}$ paths colored by red or magenta. Note that any two paths from $s_{a}$ to $t_{b}$ and from $s_{b}$ to $t_{a}$ are intersected. (Bottom-Left) From $s_{a}$ to $t_{a}$ or $t_{b}$, (Bottom-Right) from $s_{b}$ to $t_{a}$ or $t_{b}$, the numbers of paths calculated by token-passing in the “Methods” section. 1, 2, 3, 4, 6 are the numbers of reached tokens at the tips of arrows. They represent the numbers of paths which go through each edge from $s_{a}$ or $s_{b}$.

**Figure 2:**
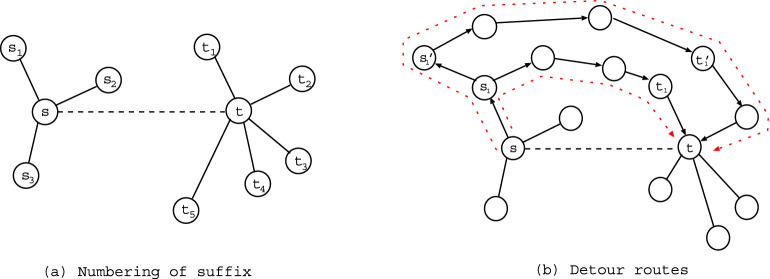
Basic process in the mapping to directed acyclic graphs from a planar network. (**a**) Parts of the connecting nearest-neighbors of *s* and *t*. Solid lines are connection edges. Dashed line represents the virtual *s*-*t* line segment. (**b**) Red dotted lines represent detour routes. $s'_{1}$ and $t'_{1}$ are the next-nearest neighbors of *s* and *t*.

We briefly explain the outline of experiments. The top in Fig. 1 shows combinations of non-intersecting paths, which we want to count the number. The colored paths by blue or green and red or magenta have no common vertices. For any pairs of source and terminal vertices denoted by *s* and *t*, we consider $k = 2, 3, \ldots$ non-intersecting paths between $s_{i} \in \partial s$ and $t_{j} \in \partial t$. Figure 2a shows that $s_{1}, \ldots , s_{k}$ and $t_{1}, \ldots , t_{k}$ are chosen by the basic process in our method explained later. Non-intersecting paths between them must pass through different vertices from $s_{1}, \ldots , s_{k}$ to $t_{1}, \ldots , t_{k}$ as start and end points, respectively, since no common vertex on the paths is necessary except *s* and *t*. Therefore, the maximum *k* is smaller number of $|\partial s|$ or $|\partial t|$. Here, $\partial s$ denotes a set of the connecting nearest-neighbors of *s*, $|\partial s|$ is the size as its degree (the number of edges emanated from *s*). Moreover, to make detour routes as shown in Fig. 2b, the neighbors can be extended from the nearest ones $\{ s_{i} \}$ or $\{ t_{j} \}$ to the next-nearest ones $\{ s'_{i} \}$ or $\{ t'_{j} \}$ (or next-next-nearest, next-next-next-nearest ones if necessary). Thus, our experiments are to find the combination number of *k* non-intersecting paths for each of $N(N-1)/2$ pairs as *s* and *t* in a given planar network with *N* vertices. Note that the number of paths from *s* to *t* is equal to that from *t* to *s* by symmetry on an (undirected) network.

We investigate non-intersecting paths for the following two test cases of realistic infrastructure networks. The first test case is a network defined by neighborhood relations of countries in Europe^18^ (see Supplementary Fig. S1 online for the visualization). It is supposed to be used for emergent transportation of relief supplies by land. Although an edge between two countries is simplified as a straight line, it actually consists of complexly connecting several roads. As a reason to simplify them, we consider that each line between two countries is corresponded to the minimum required immigration check or agreement to pass for emergent transportation. We use the two-letter codes^19^ for representing countries in Europe, although some countries are omitted in the following exceptions.

- Small countries on national border Andora, Republic of Kosovo, San Marino, Status Civitatis Vaticanae, Monaco, Liechtenstein.
- Only one edge to its connecting neighbor, no substitutive path Spain, Denmark, Portugal.
- Island countries without land continuation Cyprus, Iceland, Ireland, United Kingdom, Malta.

**Figure 3:**
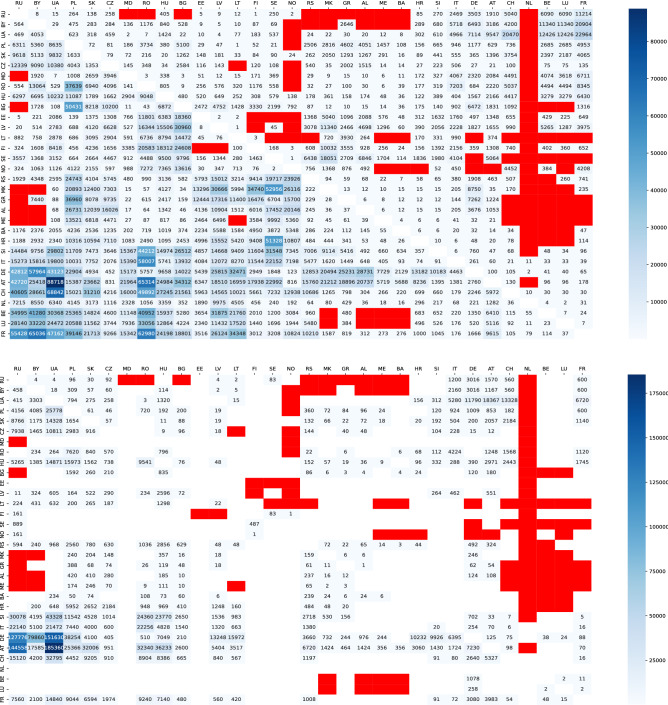
Heatmap for the combination number of (Top) $k=2$ or (Bottom) 3 non-intersecting paths on a planar network defined by neighboring countries in Europe. The right color bar indicates the number of paths from zero (white) to the maximum (blue) by gradation. The case of no substitutive path is emphasized by red. The upper triangle represents the numbers for choosing the nearest neighbors as start $s_{i}$ and end $t_{j}$ points, while the lower triangle represents the numbers for choosing the next-nearest neighbors as start $s_{i}$ and end $t_{j}$ points.

Figure 3 shows the combination numbers of (Top) $k = 2$ and (Bottom) 3 non-intersecting paths between two countries by land in Europe. In particular, as colored by dark blue, there are many combinations of $k = 2$ or 3 non-intersecting paths between a few countries in the west and the east of Europe. While as colored by red, no substitutive path exists between some countries mainly in the west and the south of Europe (see Supplementary Information online for the detail list). Between these countries, one of the counterplans for the vulnerability of connection by land may be enhancement of air-routes. In such a table of the combination numbers, each value at *ij* element in the upper triangle is smaller than that at *ji* element in the corresponding lower triangle, because the paths from $s_{i} \in \partial s$ to $t_{j} \in \partial t$ are limitted without long detour paths. Note that the diagonal parts in Fig. 3 are omitted because of the meaningless case of $s = t$.

More specifically as an example, for three non-intersecting paths from *s*:SI to *t*:FR, the following three cases of chosen neighbors are investigated.

1. $s_{1}$:HU, $s_{2}$:AT, $s_{3}$:IT, and $t_{1}$:DE, $t_{2}$:CH, $t_{3}$:IT, one combination of paths by $$\det W = \left| \begin{array}{ccc} 5 &{} 7 &{} 0\\ 2 &{} 3 &{} 0\\ 0 &{} 1 &{} 1\\ \end{array} \right| = 1$$, SI-HU-SK-CZ-DE-FR, SI-AT-CH-FR, SI-IT-FR.
2. $s_{1}$:HU, $s_{2}$:AT, $s_{3}$:IT, and $t_{1}$:BE, $t_{2}$:CH, $t_{3}$:IT, three combinations of paths by $$\det W = \left| \begin{array}{ccc} 15 &{} 7 &{} 0\\ 6 &{} 3 &{} 0\\ 0 &{} 1 &{} 1\\ \end{array} \right| = 3$$, SI-HU-SK-CZ-DE-BE-FR, SI-AT-CH-FR, SI-IT-FR. SI-HU-SK-CZ-DE-LU-BE-FR, SI-AT-CH-FR, SI-IT-FR. SI-HU-SK-CZ-DE-NL-BE-FR, SI-AT-CH-FR, SI-IT-FR.
3. $s_{1}$:HU, $s_{2}$:AT, $s_{3}$:IT, and $t_{1}$:LU, $t_{2}$:CH, $t_{3}$:IT, one combination of paths by $$\det W = \left| \begin{array}{ccc} 5 &{} 7 &{} 0\\ 2 &{} 3 &{} 0\\ 0 &{} 1 &{} 1\\ \end{array} \right| = 1$$, SI-HU-SK-CZ-DE-LU-FR, SI-AT-CH-FR, SI-IT-FR.

The above sequence of codes with hyphens represents a path of connecting vertices in the order from left to right. The total combination number is $1 + 3 + 1 = 5$, whose value is written with white background on the bottom at the cross point of row SI and column FR in the upper triangle of Fig. 3(Bottom). Each element $w_{ij}$ of path matrix *W* represents the number of paths between $s_{i}$ and $t_{j}$ in the directed graph without edges of *s*-$s_{i}$ and *t*-$t_{j}$, $1 \le i, j \le 3$. The directed graph is mapped for a given pair of *s*:SI and *t*:FR, although the detail is explained in the “Methods” section. Other cases are investigated in the same way for choosing different *s*, *t*, $s_{1}, \ldots , s_{k}$, $t_{1}, \ldots , t_{k}$, and the value of *k*.

In addition, the existing cases of more than three non-intersecting paths are mainly in UA $\sim$ PL, UA $\sim$ DE, UA $\sim$ AT, PL $\sim$ DE, and SK $\sim$ AT. Here, $s \sim t$ denotes the paths between *s* and *t* (see Supplementary Fig. S2 online for the cases of $k=4,5$). However, $k \ge 6$ paths are intersecting in our method for the first test case.

**Figure 4:**
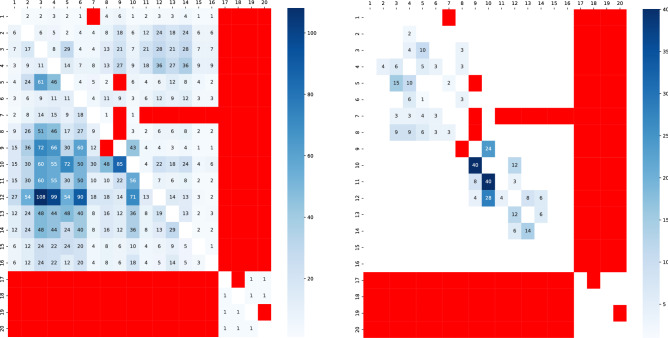
Heatmap for the combination number of (Left) $k=2$ or (Right) 3 non-intersecting paths on a backbone communication network in Japan. The right color bar indicates the number of paths from zero (white) to the maximum (blue) by gradation. The case of no substitutive path is emphasized by red. The upper triangle represents the numbers for choosing the nearest neighbors as start $s_{i}$ and end $t_{j}$ points, while the lower triangle represents the numbers for choosing the next-nearest neighbors as start $s_{i}$ and end $t_{j}$ points

The second test case is a backbone communication network connected with cables in the main island of Japan^20^. Note that the vertices are numbering from 1 to 20 according to the locations, e.g. from north to south in omitting some vertices that have only one neighbor as a common gate on paths from each of these vertices. The exact locations of vertices and the visualization should be secret for avoiding to be targets of attacks chosen by terrorists or other malice. Figure 4 shows the combination numbers of (Left) $k = 2$ and (Right) 3 non-intersecting paths between two vertices in the communication network. However, $k \ge 4$ paths are intersecting in our method for the second test case. As colored by dark blue, there are many combinations of $k = 2$ or 3 non-intersecting paths between the vertices: for $k=2$, $3{-}5$, $3{-}9$, $3{-}12$, $4{-}9$, $4{-}12$, $5 \sim 12$, $6{-}9$, $6{-}12$, $9{-}10$, $10{-}12$ in the lower triangle of Fig. 4(Left), and for $k=3$, $9{-}10$ in the upper triangle, $9{-}10$, $10{-}11$, $10{-}12$ in the lower triangle of Fig. 4(Right). While as colored by red, no substitutive path exists from each of vertices: 7, 17, 18, 19, 20, for which some sort of counterplans should be taken.

**Figure 5:**
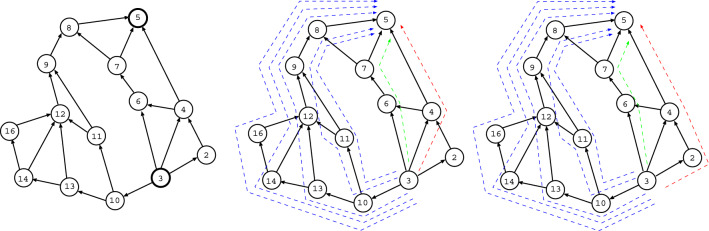
Example of $k = 3$ non-intersecting paths from 3 to 5. Three non-intersecting paths are colored by red, green, and blue. (Left) A part of directed graph, (Middle) five combinations of the paths, (Right) other five combinations of the paths. Note that only red line is different via vertex 2.

Figure 5 illustrates the combinations of three non-intersecting paths from 3 to 5. In this example, the following two cases of chosen neighbors are investigated.

1. $s_{1}$:10, $s_{2}$:6, $s_{3}$:4, and $t_{1}$:8, $t_{2}$:7, $t_{3}$:4, five combinations of paths by $$\det W = \left| \begin{array}{ccc} 5 &{} 0 &{} 0\\ 1 &{} 1 &{} 0\\ 1 &{} 1 &{} 1\\ \end{array} \right| = 5$$,
2. $s_{1}$:10, $s_{2}$:6, $s_{3}$:2, and $t_{1}$:8, $t_{2}$:7, $t_{3}$:4, five combinations of paths by $$\det W = \left| \begin{array}{ccc} 5 &{} 0 &{} 0\\ 1 &{} 1 &{} 0\\ 1 &{} 1 &{} 1\\ \end{array} \right| = 5$$.

Thus, the total combination number is $5 + 5 = 10$, whose value is written with light blue background on the top at the cross point of row 3 and column 5 in the upper triangle of Fig. 4(Right). We remark that blue paths are broken in Fig. 5, if a vertex 8, 9 or 10 is attacked and removed. However, red and green paths are remained, the connectivity between vertices 3 and 5 is sustained because of non-intersecting at the damaged point. We consider the robustness of paths as this meaning.

In this way, we have investigated the combination number of $k = 2, 3, 4, 5$ non-intersecting paths through computer simulations for realistic networks of countries by land in Europe and of backbone communication system in Japan. In particular, the tendency of many or few combinations of substitutive paths are found as follows.

- Many substitutive paths between some countries in the west and the east of Europe
- No such paths between some countries in the west and the south of Europe, when long detour paths are limited without the extension to the nearest neighbors of *s* or *t*
- Similar tendency in some parts colored by blue and red in Fig. 4 for the backbone communication network

The above results exhibit the existing of substitutive paths, as mentioned in the next section, it becomes an issue how many non-intersecting paths is usable after what types of damages.

In summary, in taking into account the construction of infrastructure networks on the surface of Earth, we have extendedly applied the counting method^21^ of non-intersecting paths based on the path matrix for a directed acyclic graph. This application provides not exact but approximate solutions for counting substitutive paths between two vertices, in adaptive mapping the directions of edges for each pair of *s* and *t* on a planar network. However, it does not give an approximation guarantee theoretically, while the origin of NP-hardness is related to that each edge has two directions possibly. Therefore there are $2^{M}$ directed graphs in the search space. For example in the test cases of countries by land in Europe with $N=36, M=74$ and a backbone communication system in Japan with $N=25, M=40$, the sizes are huge: $10^{22}$ and $10^{12}$. It is intractable to get the exact solution as the ground truth. Remember that no practical algorithm is known^16^ for counting the combination numbers of *k* non-intersecting paths. Even as approximation, it becomes possible to count them in a planar network. This is an advantage of our method at the current stage. In other words, our approach may give the first step to open the door for counting them.

## Discussion

For further studies, the following issues are considered, although the limitation of our method is still unknown.

First, to find longer detour paths, the further extension of $\{ s_{i}\}$ or $\{ t_{i}\}$ is required from the nearest neighbors. However, there is a trade off how effective is the extension and more complex computations involving with the next-next, next-next-next, $\ldots$ neighbors of *s* or *t*. For an improvement, it is a challenge to detect some excluded Z-shaped paths (see Supplementary Fig. S1 online for the visualization, e.g. CZ-PL-SK-UA or BY-PL-UA-SK) in considering how to assign directions of edges or other technical ideas related to the extension of neighbors of *s* and *t*.

Second, some directed planar graphs can exist in reality, although we assume undirected ones in this paper. If some edges are removed to be a acyclic graph, we may apply our method to such cases in satisfying the fitness condition and a homeomorphic boundary to circle^22^. It will be also a further study to perform some experiments in more complex networks, such as power-grid, gas-pipeline, water-supply, supply-chain, wireless communication system, and so on, in order to emphasize the practical effect of our method.

Third, a method for counting the number of efficient paths has been discussed in a similar but different problem setting with link costs^23^. The efficient paths are considered within a small range beyond the shortest ones. This method has a polynomial time-complexity because of having link costs. Therefore, for the quantitative evaluation, it can be not compared to our method. Although the minimizing link costs, such as distances or travel times, is one of the selections, the maintaining flows within capacities or other objectives are also possible. We wander what criteria is the optimal or sub-optimal, it is not uniquely determined in depending on a purpose of application for utilizing paths in transportation or communication. Apart from a general counting method on a planar network in this paper, the discussion involves what is desirable on what target system is considered.

Moreover, there are several robustness measures^24^, average efficiency defined by the shortest path length, algebraic connectivity related to the graph spectrum, and so on, instead of our measure of the connectivity through paths. Their analyses are different respectively. Since we aim to investigate a local property: which pairs of vertices have many or few substitutive paths, the above global measures of whole network seem to be not suitable. Even if we concentrate the connectivity through paths as the robustness, the following problems are happen. We assume that one of non-intersecting paths is emergently used as the substitutive path instead of a disconnected usual path by unspecific disasters or attacks, while as specific ones, many malicious attacks have been known: high degree attacks^25^, localized attacks (by removing a fraction of connected part)^26^, loop destruction attacks^27^, and others^28,29^ for giving strong damages to a network. It is important to investigate how many non-intersecting paths are remained after such attacks. The worst case called critical node detection (CND)^30^ is given by a set of vertices, whose removals minimize the connectivity of paths between vertices on the network. However, the CND problem becomes another NP-hard. Thus, the decreasing of the number of non-intersecting paths depends on the various types of attacks, further discussions are required through many computer simulations or theoretical analyses if possible.

## Methods

### Brief review of path matrix

We review the counting method of non-intersecting paths on a planar graph which is directed acyclic^17^. It has 2*k* boundary vertices of source and terminal, which are labeled counterclockwise as $s_{1}, s_{2}, \ldots , s_{k}$ and $t_{k}, t_{k-1}, \ldots , t_{1}$, respectively, on a homeomorphic boundary to circle. Each edge has a direction, e.g. from left to right or from top to down (see Fig. 2a again). Such a graph satisfies the fitness condition^22^:

if $P \in {{{\mathcal {P}}}}(s_{i}, t_{j})$, $Q \in {{{\mathcal {P}}}}(s_{g}, t_{h})$, $i < g$, and $j > h$, then any two paths *P* and *Q* are intersect,

where ${{{\mathcal {P}}}}(s_{i}, t_{j})$ denotes a set of paths from $s_{i}$ to $t_{j}$ on the graph. This condition can be understood intuitively for a planar graph as follows. When $s_{i}$, $s_{g}$, $t_{j}$, and $t_{h}$ are set on a circle’s circumference in the order of counterclockwise, the circle is divided into upper and lower parts by a path from $s_{i}$ to $t_{j}$. Then, since $s_{g}$ is on the lower circle’s circumference while $t_{h}$ is on the upper circle’s circumference, any path from $s_{g}$ to $t_{h}$ must be intersect (crossing) to the path from $s_{i}$ to $t_{j}$ as the border line of division.

A $k \times k$ path matrix *W* is defined for a directed acyclic graph with 2*k* boundary vertices. Each element $w_{ij}$ of *W* represents the number of paths from $s_{i}$ to $t_{j}$, which may be intersecting. For example in Fig. 1, it is given by

$$\begin{aligned} W = \left( \begin{array}{cc} 6 &{} 10 \\ 3 &{} 6 \\ \end{array} \right) , \;\;\; \det W = \left| \begin{array}{cc} 6 &{} 10 \\ 3 &{} 6 \\ \end{array} \right| = 6. \end{aligned}$$

Thus, the combination number is six for two non-intersecting paths from $s_{a}$ to $t_{a}$ and from $s_{b}$ to $t_{b}$ at the top in Fig. 1. Each element $w_{ij}$ are calculated by token-passing, such as $w_{11} = 3 + 3 = 6$, $w_{12}= 6 + 4 = 10$, $w_{21} = 1 + 2 = 3$ and $w_{22} = 3 + 3 = 6$ shown at the bottom in Fig. 1. The above matrix *W* has a property of totally positivity. In general, a matrix *W* is called totally nonnegative (or positive) if each of its minor determinants is nonnegative (or positive)^17,21^. For example, this condition is unsatisfied when $w_{kk} = 0$, then $s_{k}$ has to be connected to a path to one of $t_{1} \ldots$ or $t_{k-1}$; even if there exist one path from $s_k$ to $t_{1} \ldots$ or $t_{k-1}$ and another path from $s_{1} \ldots$ or $s_{k-1}$ to $t_{k}$, these paths are intersect. A totally nonnegative matrix and its corresponding planar graphs are theoretically associated to the analysis of symmetric functions such as Schur polynomials^17^, however the related discussion is beyond our current scope of practice. For a planar graph, its corresponding nonnegative matrix is determined uniquely, while the existing of some planar graphs is possible for a nonnegative matrix in the one-to-many relations.

Returning to the remaining subject in our proposed method as mentioned later, we consider $\{ s_{i} \}$ and $\{ t_{i} \}$ as sets of connecting neighbors of two vertices *s* and *t* at different locations. In this case, the number $| \partial s|$ or $| \partial t|$ of boundary vertices is corresponded to a degree of vertex. Since the average degree is less than six in a planar graph^31^, it is expected that *k*, $| \partial s|$, or $| \partial t|$ is a small constant. Note that the direct computation of determinant is *O*(*k*!) for any $k \times k$ matrix. However, even if *k* is large, a totally positive matrix can be factorized into a LU decomposition^17^

$$\begin{aligned} \det W = \det (LU) = (\det L) \times (\det U) = (\Pi _{i} L_{ii}) \times (\Pi _{i} U_{ii}), \end{aligned}$$

where *L* and *U* denote lower triangle and upper triangle matrices, $L_{ii}$ and $U_{ii}$ are their diagonal *i*-th elements. Then, the determinant is quickly computed with *O*(*k*). As the preprocessing, the additional computation of LU decomposition is $O(k^{3})$. We should remark that *k* is a given fixed integer, therefore the above evaluation becomes a constant multiplier for the time-complexity with respect to the size *N* or *M*.

### How to count the combination number of non-intersecting paths

We propose a mapping to directed acyclic graphs from a given planar network. The directions of edges are changeable for chosen each pair of *s* and *t*. In the mapping, we must solve (1) how to assign a direction of edge, (2) how to define a $k \times k$ path matrix *W*, and (3) how to calculate the element $w_{ij}$. We assume that $\{ s_{i} \} \cap \{ t_{j} \} = \phi$, $s_{i} \ne t_{j}$, *s* and *t* are not directly connected, otherwise the combination numbers of exceptional paths are counted in advance. For example, if there exist a direct path between *s* and *t* ($\exists$ edge (*s*, *t*)) and another path of *s*-$s_{i}$-*t* via a common node $s_{i}=t_{j}$, then instead of these exceptional two paths, the remaining $k-2$ paths from $\partial s$ to $\partial t$ are investigated after removing *s*, *t*, and $s_{i}=t_{j}$ to avoid passing through them on the other paths. In the following for the remaining paths, the connected part from $\partial s$ to $\partial t$ is mapped to a directed acyclic graph.

Let us consider $s_{i} \in \partial s$ and $t_{j} \in \partial t$ for a pair of *s* and *t* on the network. Since a path matrix *W* is a $k \times k$ square, we set $k {\mathop {=}\limits ^\mathrm{def}} \min \{ |\partial s|, |\partial t| \}$ as the smaller degree of vertex. As shown in Fig. 2a, the default suffix number from 1 to $|\partial s|$ or $|\partial t|$ is assigned according to the location of vertex in the orthogonal direction to the *s*-*t* line segment in order to satisfy the fitness condition. When two subsets of *k* vertices are chosen from $\partial s$ and $\partial t$, they are renumbered from 1 to *k* by filling missing numbers of unchosen vertices. The total combination number of non-intersecting paths is given by the sum of countings for all of the combinations ${}_{|\partial s|}C_{k} \times {}_{|\partial t|}C_{k}$ in choosing pairs of *k* vertices. In the case of choosing the next-nearest neighbors, a vertex in $\{ s'_{i} \}$ (or $\{ t'_{j} \}$) is chosen in corresponding to its connecting with the nearest neighbor $s_{i}$ (or $t_{j}$) of *s* (or *t*). Usually, $s'_{1}, \ldots , s'_{k}$ and $t'_{1}, \ldots , t'_{k}$ consist of different vertices. Here, the edges between *s* and $s_{i}$ or *t* and $t_{j}$ are ignored (removed) and not used in calculating $w_{ij}$ to avoid that they become common vertices in the non-intersecting paths.

Moreover, we consider the following greedy method without making cycles, which is slightly analogous to compass routing^32^ in computer science. In principle, there are $2^{M}$ combinations of directions for the total *M* edges. However, in order to reduce the combinations, for an edge $e_{uv}$, one of two directions between its ends *u* and *v* is temporally assigned in $\pm 90^\circ$ to the *s*-*t* line segment. It can be checked by the inner-product of these vectors $s \rightarrow t$ and $u \rightarrow v$ (or $v \rightarrow u$) based on which direction is suitable. Because the angle is in $\pm 90^\circ$, when the inner-product is positive. As exceptions, the directions from *s* to $s_{i}$ and from $t_{j}$ to *t* are implicitly assumed to shape detour routes. Figure 2b illustrates detour routes. However this method excludes Z-shaped paths with mixing of forward and backward directional edges to the *s*-*t* line segment, therefore is not exact for counting paths. It gives just an approximate solution for practical use.

**Figure 6:**
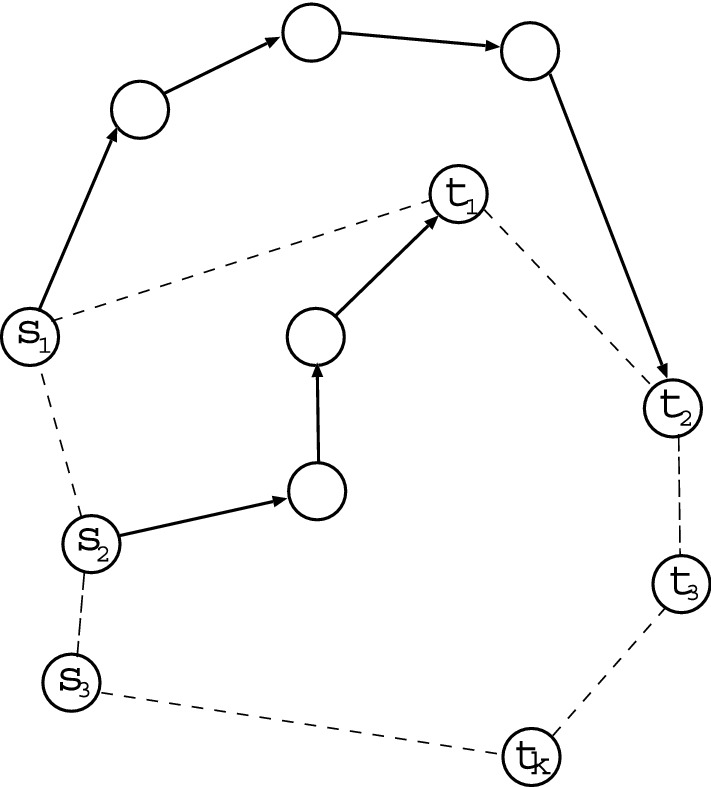
Example of an exceptional case. Solid lines are paths. Dashed virtual lines form a boundary.

In the scope for the definition of *W*, the related vertices to the paths should be included in an area whose boundary encloses the chosen pair of *k* vertices. Remember that the boundary can take any form which is homeomorphic to circle. After assigning the directions of edges according to a chosen pair of *s* and *t*, if a path from $s_{1}$ to $t_{2}$ is passing through the outside of both virtual lines $s_{1}$-$t_{1}$ and $t_{1}$-$t_{2}$ (or more than two lines) on the quadrilateral $s_{1}$-$t_{1}$-$t_{2}$-$s_{2}$ (or polygon $s_{1}$-$t_{1}$-$\ldots$-$t_{k}$-$s_{k}$-$\ldots$-$s_{2}$ ) as shown in Fig. 6, then the path-matrix is not able to be applied because of the unsatisfied fitness condition. When such an outer path is found in the above pre-checking, it is treated individually and the remaining paths that are non-intersecting with the outer one are counted, e.g. in the case of $k=2$ (or $k \ge 3$) after finding an outer path from $s_{1}$ to $t_{2}$, the remaining non-intersecting path from $s_{2}$ to $t_{1}$ is explored (or in addition other $k-2$ non-intersecting paths from $s_{3}$ to $t_{3}$ $\ldots$ from $s_{k}$ to $t_{k}$ are counted by using a path matrix).

**Figure 7:**
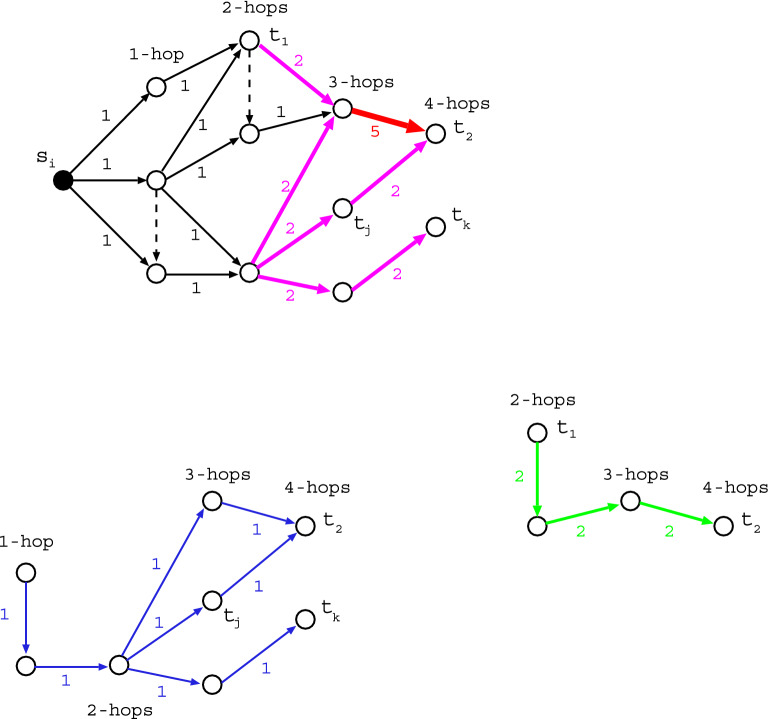
Token-passing from $s_{1}$. (Top) Primary tokens which reach at first the layered vertices by hops. (Bottom-Left) Secondary tokens with a delay of one time-step delivered from the middle vertex on 1-hop layer. (Bottom-Right) Thirdly tokens with a delay of two time-steps delivered from the top vertex on 2-hop layer. The thickness of arrow is corresponding to the number of tokens: 1, 2, or 5. Each element $w_{ij}$ is defined by the sum of primary, secondary, and thirdly tokens reached at the terminal vertices, in this case of $i = s_{1}$ and $j = t_{1}, t_{2}, \ldots$, or $t_{k}$.

On the above preparing, we calculate each element $w_{ij}$ defined as the number of paths from $s_{i}$ to $t_{j}$ which may be intersecting. It is easily obtained by using token-passing as shown in Fig. 7 according to the directions of edges assigned for each pair of *s* and *t*, since the sum of received tokens at each vertex is equal to the number of paths from the source to that vertex. Here, the received tokens are broadcasted to the vertices which are connected by its outgoing edges. The computation of a set $\{ w_{ij} \}$ of elements is evaluated as follows. From $s_{i}$ to layered vertices by hops as shown in Fig. 7(Top), the primary tokens move on *M* edges at most. In addition, the number of some edges in a same layer is *O*(*M*) at most as shown in Fig. 7(Bottom), although they are few as expected. Each of these edges makes delayed flows on *O*(*M*) edges at most by the secondary, thirdly, $\ldots$ tokens. Thus, the total time-complexity is $O(N^{2} M^{2})$ estimated from $O(N^{2})$ pairs of *s* and *t* times $O(M^{2})$ by token-passing. Moreover, the token-passing is verified from the equivalence to a polynomial-time algorithm^33^. The graph is acyclic, therefore the passing process from $s_{i}$ to $t_{j}$ is terminated eventually without keeping turn-round of tokens forever. This halting property is important to be a practically solvable problem for counting non-intersecting paths.

Finally, we summarize these processes as the outline. For each setting of $k = 2, 3, \ldots$, the combination number of *k* non-intersecting paths is given by $\det W$ in choosing subsets of *k* vertices from $\partial s$ and $\partial t$, and it is accumulated for all of the combinations of *k* vertices chosen as the subsets. We remark that the path matrix *W* and its elements $w_{ij}$ are dynamically defined according to changeable directions of edges and chosen subsets of *k* vertices for each pair of *s* and *t*.

## Supplementary Information

Supplementary Information.

## Data Availability

The data analyzed in this study are available from the corresponding author on reasonable request.
