# Supplementary material for: Practical counting of substitutive paths on a planar infrastructure network
Source: Sci Rep. 2022 Aug 29;12:14673. doi: 10.1038/s41598-022-18927-w (PMC9424307; doi:10.1038/s41598-022-18927-w)
Supplement: Supplementary file 1 — Supplementary Information. [file 41598_2022_18927_MOESM1_ESM.pdf]

Practical counting of substitutive paths on a planar infrastructure networks

Yukio Hayashi[1], and Atsushi Tanaka[2]

1: Japan Advanced Institute of Science and Technology, 2: Yamagata University

Supplementary Materials

Visualization

Figure S1 visualizes the geographic relations by land. Each vertex is located according to the longitude latitude of its capital city. Some additional vertices without two-letter codes are set to be a planar network because of the complicate shapes of national border. These additional vertices make polygonal lines corresponded to curved roads.

Detail list for Fig. 3

In particular, as colored by dark blue,

> there are many combinations of $k = 2$ or 3 non-intersecting paths between a few countries in the west and the east of Europe

as follows. Remember that $s \sim t$ denotes the paths between s and t.

(a) For $k = 2$, BY ∼ FR, UA ∼ CH, UA ∼ FR, in the upper triangle of Fig. 3(Top), and

RO ∼ PL, BG ∼ PL, LV ∼ BG, FI ∼ RO, FI ∼ BG, RS ∼ PL, RS ∼ NO, MK ∼ PL, MK ∼ LV, MK ∼ FI, MK ∼ SE, MK ∼ NO, GR ∼ PL, AL ∼ PL, AL ∼ NO, HR ∼ SE, SI ∼ UA, SI ∼ RO, SI ∼ BG, SI ∼ SE, IT ∼ RO, IT ∼ SE, DE ∼ RU, DE ∼ BY, DE ∼ UA, DE ∼ PL, DE ∼ LV, DE ∼ LT, DE ∼ MK, DE ∼ GR, DE ∼ AL, AT ∼ RU, AT ∼ BY, AT ∼ UA, AT ∼ SK, AT ∼ MD, AT ∼ RO, AT ∼ HU, AT ∼ BG, AT ∼ SE, AT ∼ MK, AT ∼ AL, CH ∼ RU, CH ∼ BY, CH ∼ UA, CH ∼ SK, CH ∼ RO, CH ∼ HU, CH ∼ BG, BE ∼ RU, BE ∼ BY, BE ∼ UA, BE ∼ PL, BE ∼ RO, BE ∼ LV, BE ∼ LT, LU ∼ RU, LU ∼ BY, LU ∼ UA, LU ∼ PL, LU ∼ RO, FR ∼ RU, FR ∼ BY, FR ∼ UA, FR ∼ PL, FR ∼ SK, FR ∼ RO, FR ∼ HU, FR ∼ LV, FR ∼ LT, in the lower triangle of Fig. 3(Top).

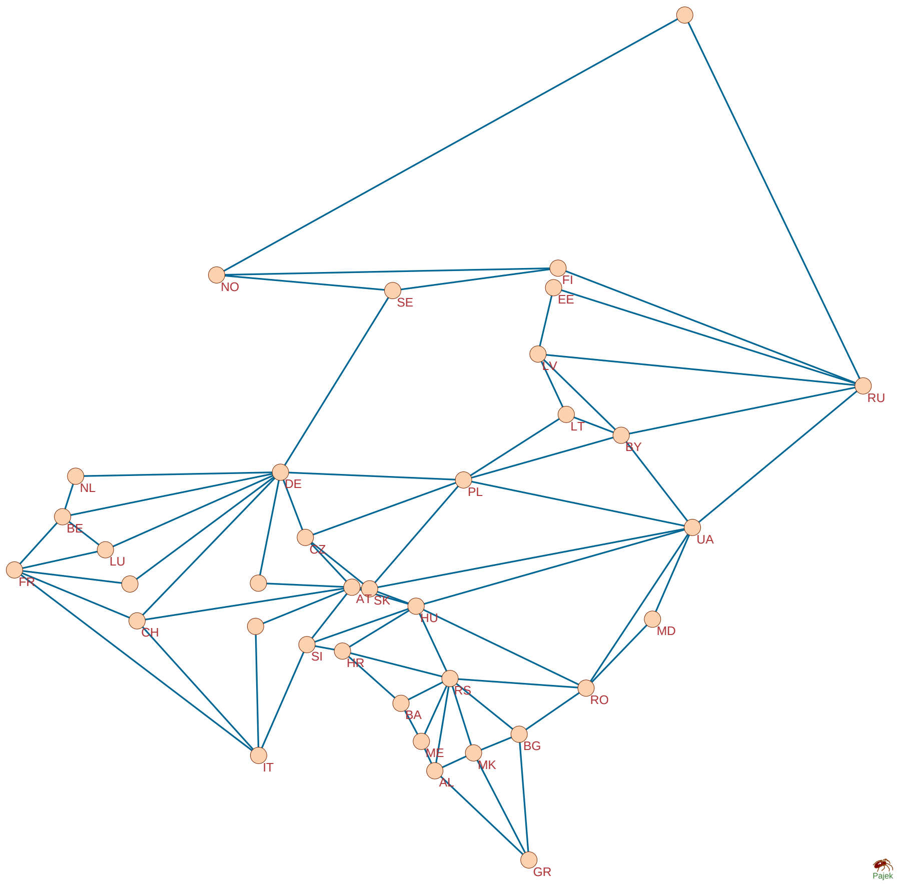

Figure S1: Relations of countries by land in Europe.

(b) For $k = 3$, RS ∼ SI, RS ∼ IT, RS ∼ DE, RS ∼ AT, BY ∼ DE, UA ∼ SI, UA ∼ DE, UA ∼ AT, PL ∼ DE, PL ∼ AT, SK ∼ AT, RO ∼ SI, RO ∼ IT, RO ∼ AT, HU ∼ SI, HU ∼ AT, in the lower triangle of Fig. 3(Bottom).

While as colored by red,

> no substitutive path exists between some countries mainly in the west and south of Europe,

such as **RU** ∼ **MD**, RU ∼ RO, **RU** ∼ **BG**, RU ∼ RS, **RU** ∼ **MK**, **RU** ∼ **GR**, **RU** ∼ **AL**, **RU** ∼ **ME**, RU ∼ BA, RU ∼ NL, BY ∼ NO, BY ∼ RS, **BY** ∼ **MK**, **BY** ∼ **AL**, **BY** ∼ **ME**, BY ∼ BA, BY ∼ NL, UA ∼ NO, UA ∼ NL, PL ∼ NO, PL ∼ NL, SK ∼ NL, CZ ∼ LT, CZ ∼ NO, CZ ∼ NL, MD ∼ NO, MD ∼ NL, RO ∼ NO, RO ∼ NL, HU ∼ NL, BG ∼ NL, BG ∼ BE, BG ∼ LU, **EE** ∼ **FI**, EE ∼ SE, EE ∼ NO, EE ∼ NL, **LV** ∼ **FI**, LV ∼ NO, LV ∼ NL, LT ∼ SE, LT ∼ NO, LT ∼ RS, **LT** ∼ **ME**, LT ∼ BA, LT ∼ DE, LT ∼ CH, LT ∼

NL, LT $\sim$ BE, LT $\sim$ LU, LT $\sim$ FR, FI $\sim$ NL, SE $\sim$ DE, SE $\sim$ CH, SE $\sim$ NL, SE $\sim$ BE, SE $\sim$ LU, SE $\sim$ FR, NO $\sim$ ME, NO $\sim$ BA, NO $\sim$ HR, NO $\sim$ DE, NO $\sim$ AT, NO $\sim$ NL, NO $\sim$ LU, RS $\sim$ NL, RS $\sim$ BE, MK $\sim$ NL, **MK $\sim$ BE**, **MK $\sim$ LU**, GR $\sim$ CH, GR $\sim$ NL, GR $\sim$ BE, GR $\sim$ LU, GR $\sim$ FR, AL $\sim$ CH, AL $\sim$ NL, **AL $\sim$ BE**, **AL $\sim$ LU**, AL $\sim$ FR, ME $\sim$ CH, ME $\sim$ NL, **ME $\sim$ BE**, **ME $\sim$ LU**, ME $\sim$ FR, BA $\sim$ NL, **BA $\sim$ BE**, **BA $\sim$ LU**, HR $\sim$ NL, HR $\sim$ BE, HR $\sim$ LU, SI $\sim$ NL, AT $\sim$ NL in the upper triangle of Fig. 3(Top and Bottom). No substitutive path remains between bold pairs in both upper and lower triangles of Fig. 3(Bottom), even for the extended choices of next-nearest neighbors as start s_i and end t_j points.

Detail for $k = 4, 5$ paths between countries by land in EU

On the next page, Figure S2 shows the combination numbers of (a) $k = 4$ and (b) 5 non-intersecting paths. The existing cases of more than three non-intersecting paths are mainly in UA $\sim$ PL, UA $\sim$ DE, UA $\sim$ AT, PL $\sim$ DE, and SK $\sim$ AT.

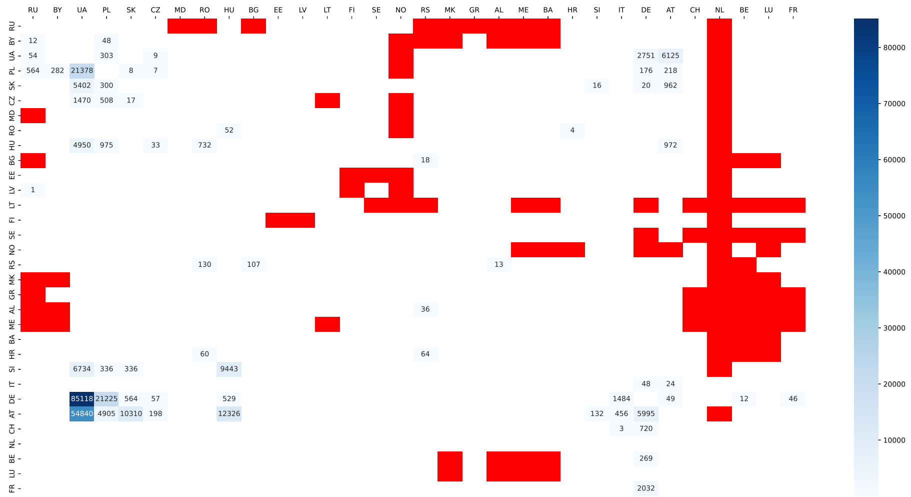

(a) 4 paths

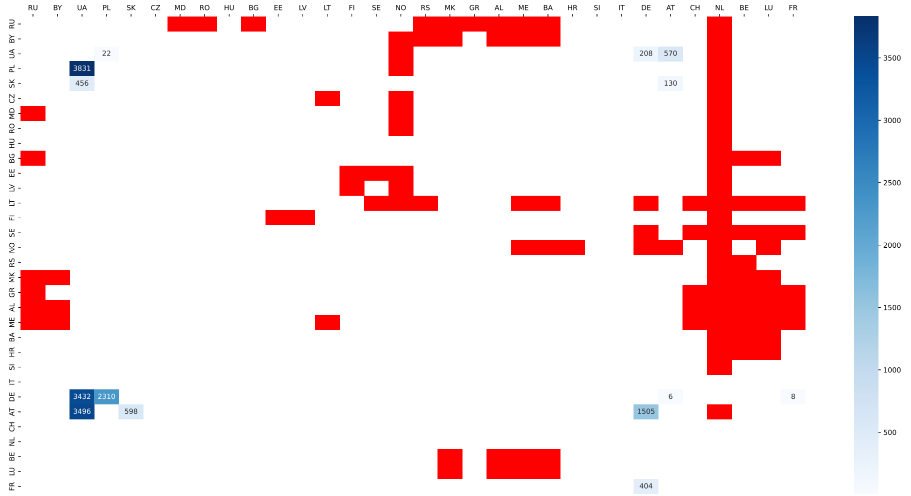

(b) 5 paths

Figure S2: Heatmap for the combination numbers of (a) four and (b) five non-intersecting paths on a planar network defined by neighboring countries in Europe. The right color bar indicates the number of paths from zero (white) to the maximum (blue) by gradation. The case of no substitutive path is emphasized by red. The upper triangle represents the numbers for choosing the nearest neighbors as start s_i and end t_j points, while the lower triangle represents the numbers for choosing the next-nearest neighbors as start s_i and end t_j points.
